# Comparison of attitudes to media representation of mental illness between journalists and mental health professionals in Russia with German-speaking countries of Switzerland, Germany, and Austria

**DOI:** 10.1177/00207640221141589

**Published:** 2022-12-28

**Authors:** Christiane Eichenberg, Lilian Strobl, Tina Jaeger, Alla Kirsha, Richard Laugharne, Rohit Shankar

**Affiliations:** 1 Institute of Psychosomatics, Sigmund Freud University, Vienna, Austria; 2 Faculty of Psychology, Sigmund Freud University, Vienna, Austria; 3 Faculty of Psychotherapy, Sigmund Freud University, Vienna, Austria; 4 Cornwall Intellectual Disability Equitable Research, University of Plymouth Peninsula School of Medicine, Truro, UK; 5 Cornwall Partnership NHS Foundation Trust, Truro, UK

**Keywords:** Media reporting, journalism, mental health awareness training, stigma, public perception

## Abstract

**Background:**

The media are an important source of information on mental health. They are often implicit in reinforcing negative stereotypes of people with mental health problems. There are no studies in German-speaking countries or Russia on media attitudes to mental health and mental health professionals’ (MHP) attitudes to the media.

**Aims:**

This study explored journalists and MHPs attitudes to mental health media reporting in the German speaking countries of Switzerland, Germany, and Austria and in Russia.

**Methods:**

A cross-sectional online internet survey, of ten Likert scale statements to ascertain perceptions of stigma, role, and training needs following the STROBE guidance was conducted among journalists and MHPs via their professional organizations. A non-discriminatory exponential snowballing technique leading to non-probability sampling was used. Descriptive statistics, Kruskal-Wallis, and a post hoc Dunn’s multiple comparisons test using Bonferroni adjustment were used to analyze data.

**Results:**

A total of 106 German-speaking and 78 Russian journalists, 109 German-speaking, and 82 Russian MHPs fully answered the survey. Journalists felt the media were more balanced about mental health than MHPs, and MHPs were wary of engagement with the media. Small minorities of journalists had engaged with mental health training, similarly few MHPs had engaged with media training, but both groups were interested in doing so in the future. Significant differences between German and Russian speaking respondents were found on a range of issues (e.g. stigmatization, image about psychotherapy, the media/MHPs, and their own role in engaging with the media/MHPs). Russians were more likely to know specialized (media/mental health awareness) training compared to German-speaking MHPs and journalists.

**Conclusion:**

There are potential opportunities to engage journalists and MHPs in training about each other’s worlds and reducing stigma toward mental illness through engaging MHPs with the media.

## Introduction

The media are an important source of information on mental health (Corrigan & Watson, 2002). They are often implicit in reinforcing negative stereotypes of people with mental health problems (Angermeyer et al., 2017; Pingani et al., 2015, 2016; Schomerus et al., 2015; Wahl, 2003). The media provide fundamental frameworks through which most people come to perceive and understand the contemporary world (Slattery et al., 2001). With suitable education and training in mental health matters for journalists, there is scope for more realistic, accurate, informative, and balanced reporting (Campbell et al., 2009). Recently national campaigns targeting negative portrayals of mental illness in the media were launched in Canada and New Zealand (Day & Page, 1986; Vaughan & Hansen, 2004). It was found that, if appropriately enlisted, the media may challenge stigma and disseminate positive mental health messages (Clement et al., 2013; Stuart, 2006). Strategies designed to reduce mental health stigma included training interventions and educational programs that addressed a range of audiences (e.g. health professionals, first responders, and the general public; Pingani et al., 2015).

Where outcomes with regard to media and mental health representation have been positive, they have illustrated that contact-based education has the capacity to reduce prejudicial cultural attitudes and improve social acceptance of people with a mental illness across different target groups and sectors (Stuart et al., 2014). Surprisingly, although cultural factors play a crucial role in the experience of stigma which shapes attitudes, beliefs, and values toward mental illness, there is limited literature focusing on the impact and importance of culture on stigma of mental illness (Abdullah & Brown, 2011). According to cross-cultural empirical research, the cultural value of individualism and collectivism is the key dimension to explain cultural differences between nations, countries, and regions (Triandis, 2001).

There are no studies in German-speaking countries or Russia on how media attitudes to mental health and mental health professionals’ (MHP) attitudes to the media may relate to one another. This study explored the attitudes of journalists toward mental health in the German speaking countries of Switzerland, Germany, and Austria and in Russia. A group of MHPs from the same countries were surveyed to identify how they see media reporting from their perspective. Differences in individualistic versus collectivistic cultures were also examined.

## Research questions

(1) What attitudes do journalists have toward mental disorders compared to MHPs?
(2) What attitudes do journalists and MHPs hold toward each other?
(3) What knowledge do journalists and MHPs hold toward bilateral awareness training?
(4) Are there differences in attitude toward media representation of mental disorders of Russian and German-speaking journalists and MHPs?

## Method

### Study design

A bespoke questionnaire was developed. The STROBE guidance for cross sectional study was followed (Supplemental Material Information 1). The prospective survey was carried out from May to September 2020. A 27 professional associations in German speaking countries and 80 in Russia were contacted with a request to forward the online questionnaire on the platform soscisurvey (https://www.soscisurvey.de) to their members.

### Study measures

The following measures were carried out:

1. Socio-demographic variables such as gender, age, country of residence, education, and specialization of work.
2. The questionnaire had two parts. Part A consisted of seven items exploring opinions of each group toward mental health rating how strongly they agreed/disagreed with each item on a 5-point Likert scale addressing research questions 1 and 2. The items were generated from a similar survey in the UK (Chapman et al., 2017). Part B included three items answered with yes/no on interest in training (‘Mental health awareness training’ for journalists and ‘Media training’ for MHPs) addressing research question 3.

### Analysis

Data was not collated systematically but gained through an exponential non-discriminative snow-balling methodology thus providing a non-probability sampling (Atkinson & Flint, 2004). Categorical data is described using count and frequency. Associations between variables were explored using a Kruskal-Wallis test and a post hoc Dunn’s multiple comparisons test using Bonferroni adjustment. The Kruskal-Wallis test and post-hoc test were employed to compare their responses to statements. Statistical significance was accepted if *p* *<* *0.05*. Data were analyzed using IBM SPSS version 27.

### Ethics

The study was approved by the Sigmund Freud University ethics committee in May 2020(GBPSRBFAO@DAU87926). All potential participants were advised that participation was voluntary, and their replies would be anonymized. A written consent was provided by all participants.

## Results

A total of 106 German-speaking journalists, 78 Russian journalists, 109 German-speaking, and 82 Russian MHPs fully answered the survey. Of the German-speaking journalists 68 (64.2%) were female and 38 (35.6%) were male. Of the Russian journalists 42 (53.8%) were female and 36 (46.2%) were male. The average age of the German-speaking journalists was 43.5 years (*SD* = 12.14), and 34.8 years (*SD* = 10.2) for the Russian journalists. Of the German-speaking MHPs surveyed 71 (65.4%) are female and 38 male (34.6%) and the average age was 52.2 years (*SD* = 12.06) whereas from the Russian MHPs 65 (79.3%) were female, 17 (20.7%) were male, and the average of the Russian MHPs was 42.4 years (*SD* = 13.4).

### German speaking countries

*Attitudes of the journalists and MHPs toward mental health, stigmatization, each other, and their own role in engaging with the media/MHPs* (Table 1)

**Table 1.** Response of German speaking MHPs and journalists to statements 1 to 7 using a Likert Scale: 1. Agree 2. Partially agree 3. Neither agree nor disagree 4. Partially disagree 5. Disagree.

Response to statements	*M*	*SD*	*U* statistics	Significance
1. Stigma is a big problem for people with mental health problems.				
MHPs	1.51	0.78	3,572	*p* ⩽ .001
Journalists	1.25	0.50		Effect size = 0.13
2. The current image of the general population about psychotherapy and MHPs needs to be improved.				
MHPs	1.53	0.77	2,539	*p* ⩽ .001
Journalists	1.96	1.08		Effect size = 0.26
3. For the general population, the media (e.g. newspapers, online and TV news, magazines) are the main source of information about mental health problems.				
MHPs	1.83	0.98	5,265	*p* = .230
Journalists	2.19	1.11		Effect size = 0.01
4. In general, journalists have a balanced media coverage of mental health issues.				
MHPs	2.81	1.02	9,495	*p* ⩽ .001
Journalists	2.47	0.96		Effect size = 0.60
5. MHPs have a key role in promoting mental health problems in the media.				
MHPs	1.86	1.07	3,721	*p* ⩽ .001
Journalists	2.02	1.09		Effect size = 0.10
6. In my professional capacity as a MHP/journalist, I would feel comfortable giving an interview to a MHP/journalist.				
MHPs	2.39	1.35	5,121	*p* = .103
Journalists	1.16	0.52		Effect size = 0.01
7. I am suspicious of the extent to which the content of the interview I have given is presented in the media.				
MHPs	2.29	1.18	2,943	*p* ⩽ .001
Journalists	2.47	1.01		Effect size = 0.21

Journalists more than MHPs responded that stigma is a major problem to those with mental illness (*p* < .001). Journalists as compared to MHPs believed that the media coverage of mental health matters was balanced (*p* < .001). MHPs, more than journalists, felt the image of MHPs and promotion of psychotherapy among the public needed improving (*p* < .001). MHPs more than journalists believed they have a key role to promote awareness of mental health matters in the media (*p* < .001). However, MHPs were wary of engagement as they had higher suspicions of media motives (*p* < .001).

*Knowledge and past participation of both groups toward the corresponding training* (Table 3)

From 106 journalists, 14 (13.2%) knew the concept of mental health awareness training of whom only 2 (1.9%) had availed it. From 109 MHPs, 18 (16.5%) knew of a media training though only 6 had undertaken it.

*Were those with no knowledge or experience toward the other profession willing to undertake relevant training?* (Table 4)

Of the 103 journalists replies, 67 (65%) were interested in taking part in it. Similarly, of the 109 MHPs, 67 (65%) were interested in partaking it. No significant differences were found between the two groups regarding their respective lack of knowledge of the concept of bespoke training or interest to participate in one.

### Russia

*Attitudes of the journalists and MHPs toward mental health, stigmatization, each other, and their own role in engaging with the media/MHPs* (Table 2)

**Table 2.** Response of Russian MHPs and journalists to statements 1 to 7 using a Likert Scale: 1. Agree 2. Partially agree 3. Neither agree nor disagree 4. Partially disagree 5. Disagree.

Response to statements	*M*	*SD*	*U* statistics	Significance
1. Stigma is a big problem for people with mental health problems.				
MHPs	3.48	1.22	3,064	*p* = .630
Journalists	3.50	1.02		Effect size = 0.001
2. The current image of the general population about psychotherapy and MHPs needs to be improved.				
MHPs	4.27	1.14	2,686	*p* = .056
Journalists	3.94	1.25		Effect size = 0.02
3. For the general population, the media (e.g. newspapers, online and TV news, magazines) are the main source of information about mental health problems.				
MHPs	3.34	1.22	2,412	*p* = .004
Journalists	3.83	1.17		Effect size = 0.05
4. In general, journalists have a balanced media coverage of mental health issues.				
MHPs	2.20	1.12	2,504	*p* = .015
Journalists	2.68	1.26		Effect size = 0.04
5. MHPs have a key role in promoting mental health problems in the media.				
MHPs	3.16	1.21	3,051	*p* = .603
Journalists	3.24	1.34		Effect size = 0.002
6. In my professional capacity as a MHP/journalist, I would feel comfortable giving an interview to a MHP/journalist.				
MHPs	3.80	1.20	1,939	*p* ⩽. 001
Journalists	4.53	0.95		Effect size = 0.14
7. I am suspicious of the extent to which the content of the interview I have given is presented in the media.				
MHPs	3.38	1.18	3,179	*p* = .946
Journalists	4.53	3.34		Effect size = 0.003

In replies to the seven statements there was noted significant difference of opinion between the two groups on three. Russian journalists felt that media played the main role in disseminating mental health information than Russian MHPs did (*p* = .004). While Media respondents felt they covered mental health matters fairly MHPs did not (*p* = .015). While media personnel felt they would be comfortable interviewing MHPs, MHPs did not share the same view (*p* < .001).

*Knowledge and past participation of both groups toward the corresponding training* (Table 4)

From 78 journalists, 38 (48.7%) knew the concept of mental health awareness training of whom only 8 (10.3%) had availed it. From 82 MHPs, 45 (54.2%) knew of a media training though only 11 (23.9%) had undertaken it.

*Were those with no knowledge or experience toward the other profession willing to undertake relevant training?* (Table 3)

**Table 3.** Response of German MHPs and Journalists to questions 1–3.

Yes/no	MHPs	Journalists
1. Do you know the concept of the training?	Yes = 18/16.5%	Yes = 14/13.2%
	No = 91/83.5%	No = 92/86.8%
2. Have you already taken part in training?	Yes = 6/5.5%	Yes = 2/1.9%
	No = 103/94.5%	No = 104/98.1%
3. Are you interested in taking part in such a training?	Media training	Mental health training
	Yes = 51/46.8%	Yes = 67/65%
	No = 58/53.2%	No = 36/35%

Of the 78 journalists, 49 (62.8%) were interested in taking part in it. Similarly, of the 82 MHPs, 72 (87.8%) were interested in partaking it. No significant differences were found between the two groups regarding their respective lack of knowledge of the concept of bespoke training or interest to participate in one.

### Comparing all the German and Russian groups with each other

A Kruskal-Wallis test provided strong evidence of a difference between the mean contribution of at least one pair of groups which showed that German-speaking journalists and MHP’s are significantly more likely to agree to almost all statements compared to Russian journalists and MHP’s who are more likely to disagree (*p* < .05; Table 5). There was very strong evidence (*p* < .001, adjusted using the Bonferroni correction) of a difference in mean ranks between the Russian and German-speaking MHPs and journalists for the following statements:

1. stigma being a problem
2. the image about psychotherapy needing improvement
3. the media being the main source of information about mental health problems
4. MHPs having a key role in promoting mental health problem

These results show that Russians were more likely to disagree to these statements than German-speaking MHPs and journalists. No significant differences were found within the groups of either German-speaking MHPs and journalists or Russian MHPs and journalists. Regarding the statement of the media generally portraying mental health issues in a balanced way Russian MHPs, were more likely to agree to this than German-speaking MHPs, or Russian journalists respectively.

(1) Comparing the attitudes of both groups toward cooperating with each other (Tables 1, 2, and 5).

Significant differences were found within and between all four groups where German-speaking journalists, felt comfortable talking to a MHP more so than Russian journalists. Further, German-speaking MHPs were significantly more likely to agree to feeling comfortable talking to a representative of the media in their professional capacity than Russian MHPs.

(2) Participation of both groups toward the corresponding training (media or mental health; Tables 3 and 4)

**Table 4.** Response of Russian MHPs and Journalists to questions 1 to 3.

Yes/ No	MHPs	Journalists
1. Do you know the concept of the training?	Yes = 45/54.2%	Yes = 38/48.7%
	No = 37/44.6%	No = 40/51.3%
2. Have you already taken part in training?	Yes = 11 / 23.9%	Yes = 8/10.3%
	No = 67 / 76.1%	No = 70/89.7%
3. Are you interested in taking part in such a training?	Media training	Mental health training
	Yes = 72/87.8%	Yes = 49/62.8%
	No = 10/12.2%	No = 29/37.3%

**Table 5.** Results of Kruskal-Wallis test and post hoc Dunn’s multiple comparisons test, showing significance of differences in Russian and German-speaking MHP’s and Journalists (R, Gs, Mhp, and Jn) regarding their responses to statements toward the representation of mental illnesses (1–7).

Groups	1. Stigma is a big problem for people with mental health problems	2. The current image of the general population about psychotherapy and MHPs needs to be improved.	3. For the general population, the media (e.g. newspapers, online and TV news, magazines) are the main source of information about mental health problems.	4. In general, journalists have a balanced media coverage of mental health issues.	5. MHPs have a key role in promoting mental health problems in the media.	6. MHP: I would feel comfortable talking to a representative of the media in my professional capacity/ Journalist: I would feel comfortable talking to a mental health practitioner in my professional capacity.	7. MHP: I am mistrustful of how an interview I give with the media may be reported/ Journalist: Mental health practitioners are mistrustful of how an interview they give with the media may be reported
	H	H	H	H	H	H	H
Jn Gs – Mhp-Gs	25.11	−30.18	−29.87	32.13	−15.83	85.7	−17.82
	*p* = .445 ns	*p* = .212 ns	*p* = .224 ns	*p* = .145 ns	*p* = 1 ns	p < .001***	*p* = 1 ns
Jn Gs – Mhp R	166.60	−157.56	−115.41	28.95	−106.3	−168.12	−96.01
	*p* < .001***	p < .001***	*p* < .001***	*p* = .356ns	*p* < .001***	*p* < .001***	*p* < .001***
Jn Gs – Jn R	−171.76	−176.47	−150.56	−16.91	−109.3	−215.04	−100.13
	*p* < .001***	*p* < .001***	*p* < .001***	*p* = 1 ns	*p* < .001***	*p* < .001***	*p* < .001***
Mhp Gs- Mhp-R	−141.5	−127.38	−85.54	61.08	−90.5	−82.42	−78.2
	*p* < .001***	*p* < .001***	*p* < .001***	*p* < .001***	*p* < .001***	*p* < .001***	*p* < .001***
Mhp Gs- Jn R	−146.65	−146.3	−120.69	15.22	−93.49	−129.34	−82.31
	*p* < .001***	*p* < .001***	*p* < .001***	*p* = 1ns	*p* < .001***	*p* < .001***	*p* < .001***
Mhp R – Jn R	−5.16	18.91	−35.15	−45.87	−3.09	−46.92	−4.12
	*p* = 1 ns	*p* = 1 ns	*p* = .207 ns	*p* = .033*	*p* = 1 ns	*p* = 0.027*	*p* = 1 ns

*Note*. Explanation of symbols *H* test value, ns = non-significant; *p* = significance level.

* *p* < .05. ***p < .001.

It was found that more Russians took part in the training compared to the German sample with 11 (23.9%) Russian MHPs compared to 6 (5.5%) German-speaking MHPs having taken part in the media training. From the Russian Journalists 8 (10.3%) compared to 2 (1.9%) Journalists took part in the mental health awareness training.

(3) Interest in media awareness or mental health training (Tables 3 and 4)

In the German-speaking sample, 91 (83.5%) MHPs were unaware of the concept of the training, 51 (49.5%) were willing to take part, 52 (50.5%) were not. From the German-speaking journalists 92 (86.8%) were unaware of the concept with 67 (64.4.1%) interested and 37 (35.6%) were not. In the Russian sample, 40 (51.3%) journalists were unaware with 49 (70%) interested and 21 (30%) not interested. From the Russian MHPs, 37 (44.6%) were unaware of the concept of the training with 50 (70.4%) interested and 21 (29.6%) uninterested.

## Discussion

This study suggests in both German speaking countries and in Russia journalists have more confidence that media reporting of mental health issues is balanced than do mental health professionals (MHPs). However, MHPs are wary of engaging with the media.

The journalists were fairly ignorant of mental health training and few had received it, and similarly few mental health professionals were aware of media training and even fewer had received it. Interestingly there was more awareness of the appropriate training and engagement in Russia than in the German speaking countries.

In both cultures journalists and MHPs were interested in engaging with the appropriate training which seems positive. MHPs were more positive about media training in Russia.

In our study, the samples of German-speaking and Russian journalists and MHPs differ in terms of their gender and age characteristics. We found that Russian media professionals are less likely to agree that there is a stigma regarding mental health compared to German-speaking journalists and MHPs. This could be explained due to cultural differences between individualistic and collectivistic cultures.

A comparison study of Russian and British attitudes toward mental health problems in the community (Shulman & Adams, 2002) revealed that the British sample was significantly more tolerant than the Russian sample. For example, the Russian sample was less likely to identify dementia as a mental disorder. Nevertheless, the British sample chose medically related help significantly more than the Russians. Further, significant associations could be found between factors such as education and familiarity with mental illness and tolerance within the British group. This study also found that observed differences in terms of historic, political, and cultural perspectives. Another review analyzed the relevant policy documents, special programs, laws, and scientific literature of Russia where results indicated that on a discursive level the MHC delivery system in contemporary Russia is developing toward more modern models and principles (Kolpakova, 2019). They found a fairly constant ‘tendency to ignore’ important topics such as stigma, social inclusion, and independent living of patients. The review established that the Russian MHC system is dominated by psychiatrists, and that cooperation with other specialists in state care and health professionals from private practices and NGOs is not common. Findings of these studies could go some way in explaining the background underpinnings for the differences identified in our study in attitudes to mental health between Russian and German groups.

A systematic review found that culture factors (e.g. Collectivism, Confucianism, face concern and familism, religion, and supernatural beliefs) contributed to people’s stigmatizing behaviors and attitudes toward persons with mental illness, their relatives and mental health professionals (Pang et al., 2017). A systematic review found that persons with mental illness and their relatives in collective societies have to struggle with more severe and widespread stigma than their counterparts in Western societies (Ran et al., 2021). Interestingly, Russian MHPs and Journalists do not seem to perceive stigma to be a problem.

Similar to our German-speaking sample, a UK study found that three-quarters of mental health service users reported that media coverage of mental health was unfair, negative, and unbalanced, and indicated that the media coverage had had a negative effect on their own mental health (Ferriman, 2000). Another UK study comparing journalists to MHPs showed that the majority of both professional groups agreed with the need to be sensitive to report mental health issues and recognized the importance of their respective roles in influencing the public (Chapman et al., 2017). This is at variance with the German-speaking sample whereas the Russian sample were more likely to disagree.

Several factors limit the generalizability of the study findings thus they need to be viewed with caution, as the low sample size limits the strength and interpretation of the results. Further studies need to elaborate more in what condition these cultural factors or values contribute to reducing stigma and helping those with mental illnesses.

There is one very important positive message from our findings. Though few had received bespoke training there was interest in all four groups in engaging with this training. The similar study in the UK found that MHPs who had undergone media training were more positive about engaging with the media (Chapman et al., 2017). If MHPs and the media had closer contact and greater understanding of each other, it is possible that the general public would receive better public education on mental health matters. It has been indicated that such kinds of education through the media have adequate influences to shape public attitude and perception toward mental illness (Luo et al., 2018).

## Supplemental Material

sj-docx-1-isp-10.1177_00207640221141589 – Supplemental material for Comparison of attitudes to media representation of mental illness between journalists and mental health professionals in Russia with German-speaking countries of Switzerland, Germany, and Austria Click here for additional data file. Supplemental material, sj-docx-1-isp-10.1177_00207640221141589 for Comparison of attitudes to media representation of mental illness between journalists and mental health professionals in Russia with German-speaking countries of Switzerland, Germany, and Austria by Christiane Eichenberg, Lilian Strobl, Tina Jaeger, Alla Kirsha, Richard Laugharne and Rohit Shankar in International Journal of Social Psychiatry
